# Adjuvant Radiotherapy After Minimally Invasive Surgery in Patients With Stage IA1-IIA1 Cervical Cancer

**DOI:** 10.3389/fonc.2021.690777

**Published:** 2021-07-26

**Authors:** Yi-xiu Gan, Qing-hua Du, Jian Li, Ye-ping Wei, Xu-wei Jiang, Xue-mei Xu, Hai-ying Yue, Xiang-de Li, Hui-jun Zhu, Xue Ou, Qiu-lu Zhong, Dan-jing Luo, Qian-fu Liang, Yi-ting Xie, Qiang-qiang Zhang, Ge-li Li, Yuan-ting Shang, Wen-qi Liu

**Affiliations:** ^1^ Department of Radiation Oncology, Second Affiliated Hospital of Guangxi Medical University, Nanning, China; ^2^ Department of Gynecology, Second Affiliated Hospital of Guangxi Medical University, Nanning, China

**Keywords:** adjuvant radiotherapy, minimally invasive surgery, laparoscopic hysterectomy, cervical cancer, open surgery, survival

## Abstract

To estimate whether adjuvant radiotherapy is necessary for patients with stage IA1-IIA1 cervical cancer after laparoscopic hysterectomy, 221 patients were retrospectively analyzed. Sixty-two of them were treated with laparoscopic hysterectomy and adjuvant radiotherapy (group A), 115 underwent open surgery (group B) and 44 received laparoscopic hysterectomy alone (group C). Results showed that the 3-year local recurrence-free survival (LRFS) rates of group A, B and C were 98.4%, 97.4% and 86.4%, respectively. The LRFS rates of group A and B surpassed C (A *vs*. B, p=0.634; A *vs*. C, p=0.011; B *vs*. C, p=0.006). The inter-group differences of 3-year overall survival (OS) and distant metastasis free survival (DMFS) were not statistically significant. In subgroup analysis of stage IB disease, the 3-year LRFS rates of group A, B and C were 100%, 98.8% and 83.1%, the 3-year OS rates of group A, B and C were 100%, 98.9% and 91.5%, respectively. The 3-year LRFS and OS rates of group A and B were significantly superior to group C (p<0.05). Our findings suggest that adjuvant radiotherapy can reduce the risk of recurrence for women with early-stage cervical cancer after laparoscopic hysterectomy and bring survival benefits for patients with stage IB disease.

## Introduction

According to National Comprehensive Cancer Network (NCCN) clinical guidelines, postoperative adjuvant radiotherapy is generally not required for stage IA1~IB cervical cancer patients if there are no high-risk factors (such as lymph-node involvement, nerve invasion, and large tumor) or the intermediate risk factors do not meet the Sedlis criteria. Recently, A recent study by Ramirez, a highly noteworthy phase III study, was published in the October 2018 New England journal of medicine, which found that the 4.5-year disease-free survival and 3-year tumor-free survival in the minimally invasive surgery group were significantly lower than those in the open surgery group, and the risk of death or recurrence in the minimally invasive group was 3.74 times higher than that in the open surgery group (1). A retrospective study by Melame et al. had similar results. It can be speculated that minimally invasive surgery may bring the risk of local recurrence (2). Therefore, we speculate that minimally invasive hysterectomy for early cervical cancer carries a risk of local failure, it is worth studying whether additional postoperative radiotherapy is needed for these patients with minimally invasive surgery.

## Methods

From January 2013 to December 2016, a total of 221 patients with early-stage squamous-cell carcinoma, adenocarcinoma, or adenosquamous carcinoma of cervical cancer admitted to our institution. Preoperative medical imaging examination (Chest/abdominal/pelvic CT, neck/chest/abdomen/pelvic/inguinal PET-CT and pelvic MR) and postoperative pathology were retrospectively reviewed to make sure that all the enrolled women had “low-risk”early-stage cervical cancer which was defined as: patients have one or more intermediate-risk factors after surgery, but the combination of intermediate-risk factors did not meet the Sedlis criteria (3–5). The clinical data of eligible patients with stage IA1 (lymph-vascular invasion)-IIA1 cervical cancer were retrospectively analyzed. All patients had an Eastern Cooperative Oncology Group (ECOG) performance-status score of 0 or 1. Exclusion criteria included a history or contraindication to radiotherapy; the advanced stage cervical cancer; absence of severe mental disorders or severe diseases of heart, liver, lung, kidney; the existence of high-risk factors (lymph-node involvement, para-uterine invasion, and positive vaginal resection margin). Patients were also excluded if the postoperative pathologic risk factors meet the Sedlis Criteria of the latest version of NCCN Guidelines (Version 1.2020). According to different treatment approaches, patients were assigned to different groups. The first group underwent laparoscopic hysterectomy combined with postoperative radiotherapy (group A, n=62), the second group only received open surgery (group B, n = 115), and the third group received laparoscopic hysterectomy alone (group C, n = 44). The median age was 47 years (24-69 years). Patients were re-staged based upon International Federation of Gynecology and Obstetrics (2018 FIGO) Surgical Staging of Cancer of the Cervix Uteri (2018) (6).

Enrolled patients received open surgery or laparoscopic hysterectomy. Rigorous preoperative discussion and evaluation before surgery were proceeded to ensure that appropriate surgical techniques were used during minimally invasive surgery or open surgery. Different classes of radical hysterectomy were performed according to patients’ different FIGO stages. Part of patients with FIGO IA1 stage (lymph-vascular invasion) and IA2 to IB1 stage diseases received type B radical hysterectomy defined in Querleu–Morrow classification while some of them underwent type C1 radical hysterectomy. Women with stage IB2-IIA2 were primarily treated with type C1 radical hysterectomy, and type C2 radical hysterectomy was performed only when the anatomical situation was very unclear, with the aim of preserving the pelvic autonomic nerve as much as possible and obtaining the most appropriate surgical margin to achieve the radical cure. Before the surgery, Patients with stage IA1 (Lymphovascular Invasement), IB1-IB2 and IIA1 were injected with tracer to find sentinel lymph nodes for sentinel lymph node biopsy (SLNB). Cryopathological examination was performed during the operation, and continuous sections and immunohistochemical staining were performed after the operation. Pelvic lymphadenectomy was determined according to intraoperative pathology. Due to the characteristic of station-by-station metastasis of lymph nodes in cervical cancer, low abdominal para-aortic lymph node dissection was performed in patients with common iliac lymph node metastasis. For patients with large local tumor size (IB3), para-aortic lymphatic dissection was also performed. Any suspected lymph nodes were also removed. If SLN tracing failed, pelvic lymph node dissection was required. The results showed that at least one SLN was identified in 91% of cases (201/221) and optimal (bilateral) SLN localization was achieved in 75% of cases. Para-aortic lymph node dissection was performed in 10% of the patients, 8 of whom were patients with a large local mass(≥4cm).

In group A and group C, Uterine manipulator was used for better exposure of the surgical field and operating easier. Under laparoscopy, the round ligament, the ovarian proper ligament and the fallopian tube isthmus (or the pelvic funnel ligament) were cut off and parametrial tissue was pushed aside step by step according to the conventional procedures. The vaginal wall was cut annularly along the edge of uterine manipulator. And then the specimens were removed and the vaginal stump was sutured under laparoscopy.

The patients of group A received post-operative radiotherapy carried out by intensity modulated radiotherapy (IMRT). According to the consensus guidelines of the Radiotherapy Oncology Group (RTOG) 0418 and its atlas, the clinical target volume (CTV) consisted of central vaginal CTV and a regional lymph node CTV, includes proximal vaginal, paraginal tissues, the internal and external iliac, and anterior sacral lymph nodes. The planned target volume (PTV) is generated by homogeneous expansion of CTV at 7mm. Plan design based on anatomical boundaries. These boundaries are: Superior-L5/S1; Lower-bottom of the obturator foramen; the lateral edge of the pelvis was 2 cm, which was adjusted according to the vascular contour. The first 5mm of the symphysis pubis should be adjusted according to the vascular contour; and the back-S2/S3. The organs at risk (OAR) profile include bladder, bowel cavity, rectum, femur head, and other normal tissues. The prescription dose of PTV is 45-50 Gy, 1.8-2.0 Gy per fraction 5 times a week carried out in 5 weeks. The goal of plan for IMRT is to obtain 95% of the prescribed dose to cover 100% of PTV, with the maximum dose not exceeding 110%. The maximum dose of OAR limiting dose defined by 2% (D2%) of the maximum tissue dose was 50 Gy. Supplementary restriction V50 Gy (volume exposed to radiation of 50 Gy): rectum < 40%, bladder < 50%, femoral head < 5%, V20Gy < 30% and V30Gy < 20% for intestine. None of the patients received concurrent chemotherapy.

SPSS 24.0 statistical software was used for data analysis. The primary outcome was 3-year local recurrence-free survival (LRFS) rates. The secondary endpoints were 3-year overall survival (OS) and 3-year distant metastasis free survival (DMFS) rates. Survival rates were used to calculate by Kaplan-Meier method. Cox regression analysis was used to estimate prognostic factors. And the statistically significant P value was a two-tailed P value less than 0.05.

## Results

### Characteristics of Patients

The median age of patients was 47 years (range, 24-69 years). All patients were restaged according to FIGO 2018 and whose previous diagnosis was stage IA-IIa cervical cancer but had positive lymph nodes were excluded from our analysis. The clinical baseline characteristics of age, tumor size, histologic subtypes, stage of disease and ECOG performance-status score were not statistically different between-groups (**Table 1**).

**Table 1:** Baseline characteristics of patients.

	No. of patients (%)			P-value
	Group A (n = 62)	Group B (n = 115)	Group C (n = 44)	
Age (years)				
Median (range)	50 (27-64)	47 (24-64)	49 (24-69)	0.277
≤47	30 (48.4)	63 (54.8)	18 (40.9)	
>47	32 (51.6)	52 (45.2)	26 (59.1)	
Tumor size (cm)				0.810
≤2	16 (25.8)	28 (24.3)	9 (20.5)	
>2	46 (74.2)	87 (75.7)	35 (79.5)	
Stage of disease				0.106
IA1 (lymphovascular invasion)	4 (6.5)	8 (7.0)	2 (4.5)	
IA2	2 (3.2)	8 (7.0)	3 (6.8)	
IB1	34 (54.8)	79 (68.7)	35 (79.5)	
IB2	14 (22.6)	10 (8.7)	2 (4.5)	
IB3	2 (3.2)	5 (4.3)	1 (2.3)	
IIA1	6 (9.7)	5 (4.3)	1 (2.3)	
Histologic subtype - no. (%)				0.150
Squamous-cell carcinoma	50 (80.6)	90 (78.3)	34 (77.3)	
Adenocarcinoma	10 (16.1)	25 (21.7)	8 (18.2)	
Adenosquamous carcinoma	2 (3.2)	0 (0)	2 (4.5)	
ECOG performance-status score				0.840
0	58 (93.5)	106 (93.0)	42 (95.5)	
1	4 (6.5)	8 (7.0)	2 (4.5)	

### Primary Outcomes

**Figure 1:**
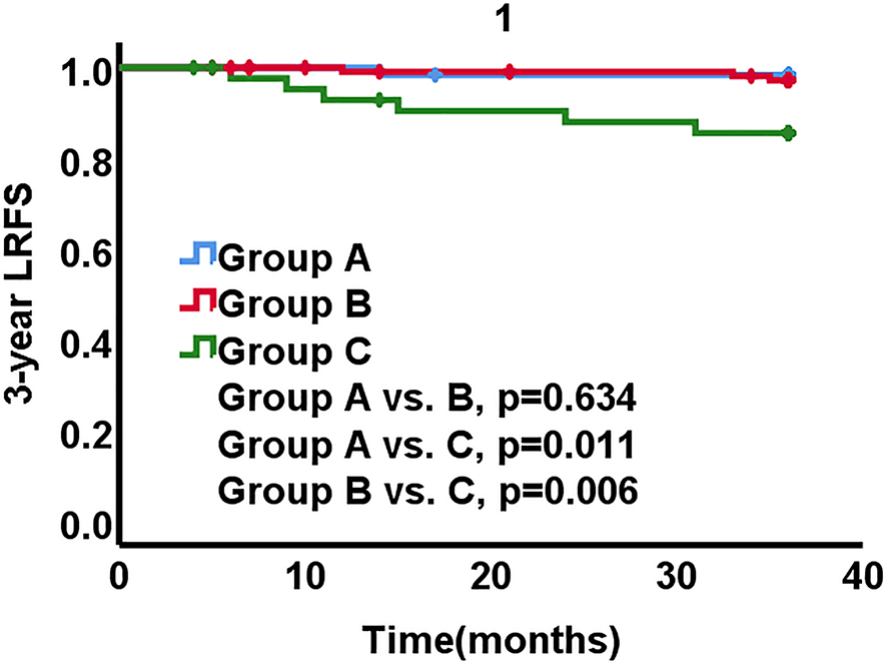
Local recurrence-free survival (LRFS) stratified by three groups. Group A, laparoscopic hysterectomy and adjuvant radiotherapy; Group B, open surgery; Group C, laparoscopic hysterectomy alone.

With a median follow-up time of 58.33 months (range, 56.90 to 59.76 months), the 3-year LRFS, DMFS and OS rates for all patients were 95.5%, 96.4% and 96.4%, respectively. A total of 10 relapses occurred in 3 years for all patients. There was one patient who encountered local failure and the 3-year LRFS rates were 98.4% in group A. Three patients encountered pelvic or vaginal recurrences and the 3-year LRFS rates were 97.4% in group B. There were 5 patients suffered from the local recurrences and the 3-year LRFS rate was 86.4% in group C, which was significantly lower than that in group A and B (A *vs*. B, p = 0.634, A *vs*. C, p = 0.011, B *vs*. C, p = 0.006) (**Table 2** and **Figure 1**).

**Table 2:** Incidence and pattern of cervical cancer failures in the three groups.

Pattern of failures	No. of patients (%)		
	Group A (n = 62)	Group B (n = 115)	Group C (n = 44)
Total failures	4 (6.5)	5 (4.3)	6 (13.6)
Pelvis recurrence	0	2 (1.7)	2 (4.5)
Vaginal vault	0	1 (0.8)	3 (6.8)
Distant metastasis	3 (4.8)	2 (1.7)	
Pelvic recurrence +di stant metastasis	1 (1.6)		1 (2.3)

### Secondary Outcomes

**Figure 2:**
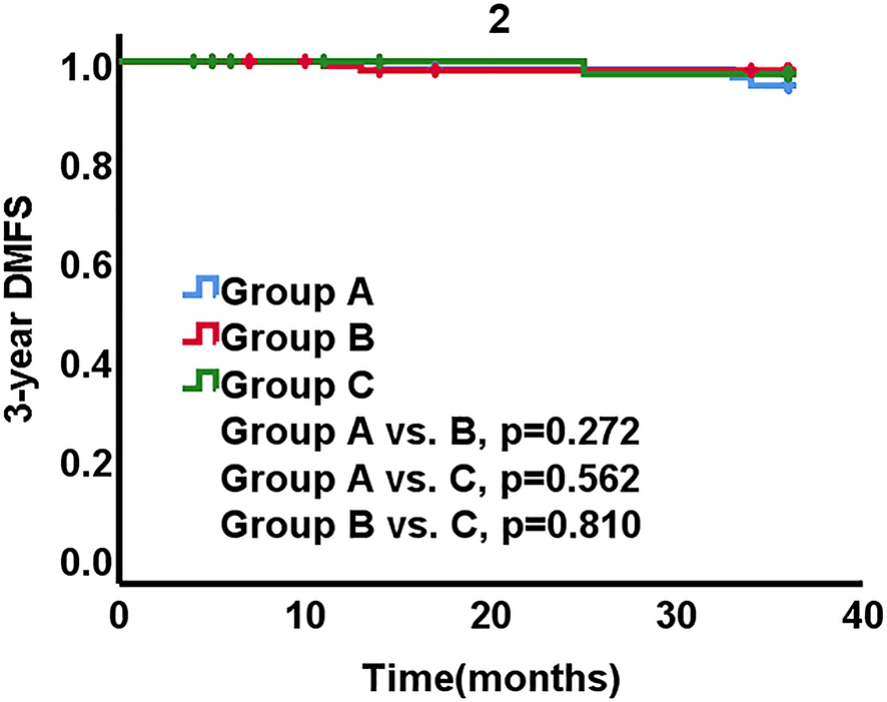
Distant metastasis free survival (DMFS) stratified by three groups.

A total of 7 patients developed lung or bone metastases during 3 years (**Table 2**). Four of them were in group A, 2 were in group B, 1 was in group C. The 3-year DMFS rates were 93.5% in group A, 98.3% in group B, 97.7% in group C, respectively. There was no statistically significant between-group difference (A *vs*. B, p = 0.123, A *vs*. C, p = 0.381, B *vs*. C, p = 0.810) (**Figure 2**).

**Figure 3:**
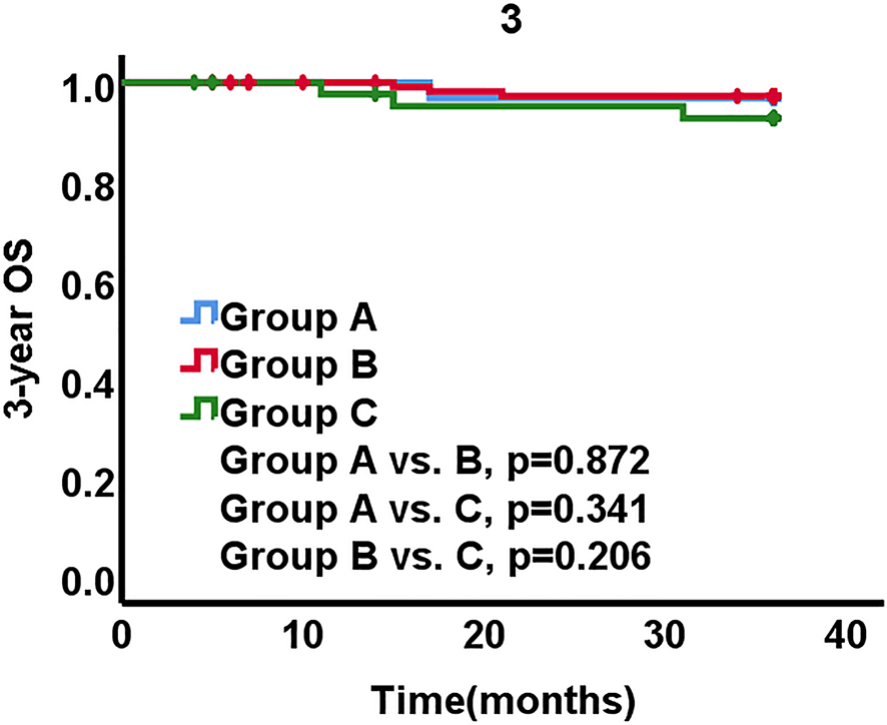
Overall survival (OS) stratified by three groups.

Of the 221 patients, 8 patients died within 3 years, all of whom were tumor-related deaths. The 3-year OS rates were 96.8% in group A, 97.4% in group B, and 93.2% in group C, respectively. The inter-group differences were not statistically significant (A *vs*. B, p = 0.872, A *vs*. C, p = 0.341, B *vs*. C, p = 0.206) (**Figure 3**).

### Subgroups Analysis

In exploratory subgroup analysis of the different stages, we compared the LRFS, DMFS and OS rates across the subgroup of stage IA disease, the subgroup of stage IB1-IB3 disease and the subgroup of stage IIA1 disease, respectively.

In subgroup of stage IA disease, there was one patients (6.2%) encountered recurrence and death from cervical cancer in group B, the 3-year LRFS and OS rates were both 93.8%. There were not any recurrences and death occurred in group A and group C. There was no statistically significant between-group difference on 3-year LRFS and OS (group A *vs*. B, p=0.540, group B *vs*. C, p=0.576). No patients with stage IA disease had distant metastasis.

**Figure 4:**
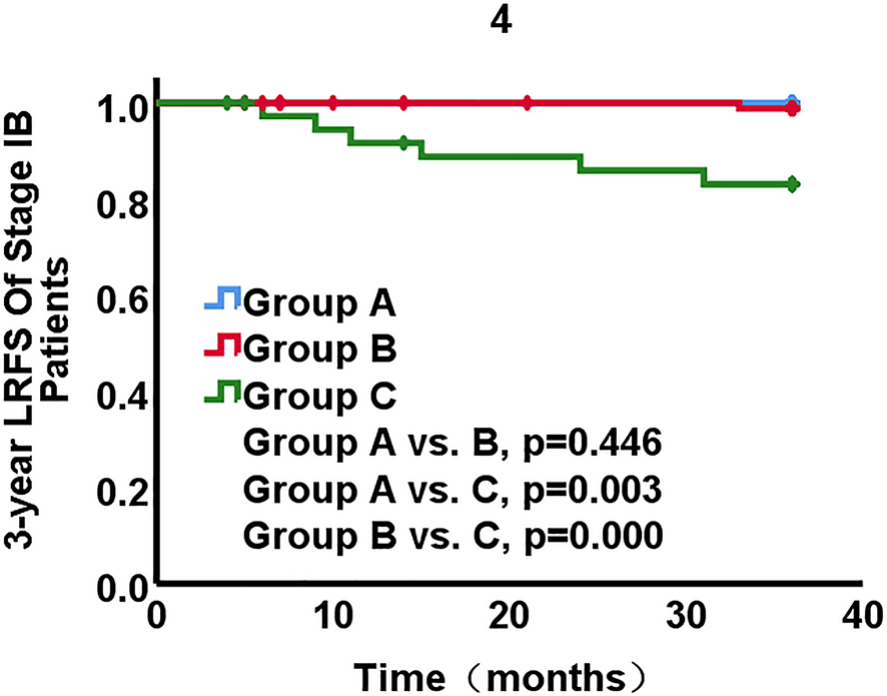
Local recurrence-free survival (LRFS) stratified by three groups for patients with stage IB1-IB3 disease.

**Figure 5:**
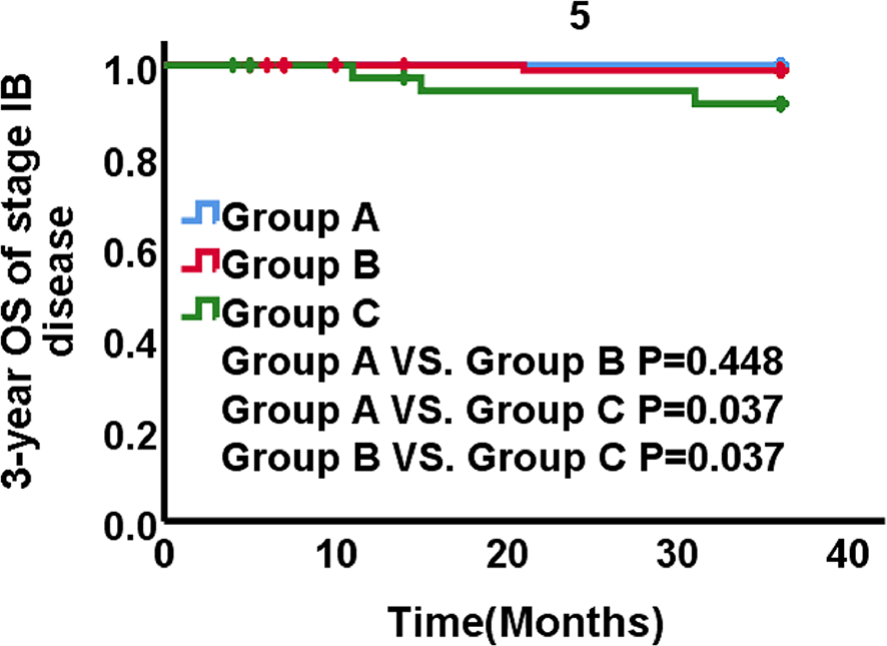
OS stratified by three groups for patients with stage IB1-IB3 disease.

In subgroup of stage IB1-IB3 disease, no recurrences and death occurred in group A. Recurrence occurred in one patients in group B and six in group C. The 3-year LRFS was 98.8% in group B and 83.1% in group C, respectively. The rates of LRFS significantly differed between the three groups (group A *vs*. B, p=0.446; group A *vs*. C, p=0.003; group B *vs*. C, p=0.000) (**Figure 4**). The 3-year DMFS rates were 96.0% in group A, 98.9% in group B, 97.0% in group C. The inter-group differences of DMFS were not different from each other (group A *vs*. B, p = 0.276, group A *vs*. C, p = 0.829, group B *vs*. C, p = 0.483). The 3-year OS rates were 100% in group A, 98.9% in group B, 91.5% in group C. The significant differences existed between groups (group A *vs*. B, p = 0.448, group A *vs*. C, p = 0.037, group B *vs*. C, p = 0.037) (**Figure 5**). The LRFS and OS benefits were observed in patients with cervical cancer stage IB1-IB3.

In subgroup of stage IIA1 disease, two patients relapsed, one of them was in group A, another was in group B. Two women had distant metastasis, one of them was in group A and another was in group B (1/5). In terms of OS, a total of three patients died for the disease, two patients were in group A, and one was in group C. The 3-year LRFS, DMFS and OS rates did not differ significantly between the three approaches (p>0.05).

### Prognostic Factors

Univariate analysis suggested that patients underwent laparoscopic hysterectomy alone had a higher rate of local recurrence than patients received laparoscopic hysterectomy combined with adjuvant radiotherapy or open surgery (hazard ratio for local recurrence, 11.39; 95% CI, 1.36 to 95.50), Multivariate analysis found that a difference remained after the adjustment for, ECOG performance-status score, stage of disease, age, and lymph-vascular invasion (LVSI) (hazard ratio for disease local recurrence from cervical cancer, 12.27; 95% CI, 1.34 to 112.58). Univariate analysis suggested that deep stromal invasion (DSI) was an independent risk factor for local recurrence (hazard ratio, 3.48; 95% CI, 1.17 to 10.37), multivariate analysis found that a difference remained with the adjustment for ECOG performance-status score, stage of disease, age, and different approaches (hazard ratio for local recurrence, 4.00; 95% CI, 1.07 to 14.99).

### Toxicities of Postoperative Radiotherapy

No grade 3 or 4 acute adverse reactions occurred in patients received postoperative radiotherapy. The incidence of grade 1 and 2 adverse reactions of bladder were 12.9% and 4.8%, respectively. The rates of grade 1 and 2 acute gastrointestinal adverse reactions were 27.4% and 9.7%, respectively. The incidence of grade 1 and grade 2 hematologic toxicities were 33.9% and 8.1%, respectively. No acute adverse reactions were observed in the femoral head (**Table 3**).

**Table 3:** The incidences of acute adverse reactions of group A.

Acute adverse reactions	Grade I n (%)	Grade II n (%)
Intestinal reaction	17 (27.4)	6 (9.7)
dermatitis	5 (8.1)	0
Bladder reaction	8 (12.9)	3 (4.8)
Hematologic toxicity	21 (33.9)	5 (8.1)

## Discussion

In this retrospective analysis, women undergoing laparoscopic hysterectomy combined with adjuvant radiotherapy or open surgery for early-stage cervical cancer had higher 3-year LRFS rates than patients who received laparoscopic hysterectomy alone. For patients with early-stage cervical cancer without any high risk factors after surgery, a prospective study conducted by Delgado et al. (4) found that lymph-vascular space invasion (LVSI), deep stromal invasion (DSI) 、and tumor size ≥2cm (TS≥2cm) were the risk factors for recurrence. And then they put forward the classic standard of adjuvant treatment for early stage cervical cancer: there are at least two intermediate risk factors (LVSI(+)、DSI>1/3、TS≥2 cm). However, this trial were carried out from March 1981 to February 1984, all patients undergo radical hysterectomy while laparoscopic hysterectomy had not been used. The evaluation of postoperative risk factors was limited in patients with squamous cell carcinoma. Sedlis et al. then combined intermediate risk factors to form the Sedlis standard (5). However, according to the study by Ryu et al. (7), the sensitivity of the Sedlis criteria was only 50%, and its scoring method was too complicated and inconvenient for clinical application. In short, there is still controversy regarding the indications for postoperative adjuvant treatment in early stage cervical cancer with intermediate risk factors. Although the NCCN guidelines (8) recommend the use of Sedlis criteria, different countries and regions still use different standards.

In 1989, Reich (9) reported the world’s first laparoscopic hysterectomy. With the continuous improvement of surgical methods, the clinical benefits of laparoscopic hysterectomy for cervical cancer have been affirmed by many studies. Study by Nam et al. (10, 11) compared the survival of patients with early stage cervical cancer between laparoscopic radical hysterectomy group and open radical hysterectomy group. They found that compared with open radical hysterectomy (n = 263), laparoscopic radical hysterectomy (n = 263) did not have higher risks of recurrence [hazard ratio (HR) =1.28; 95% confidence interval (CI) 0.62–2.64] or death (HR = 1.46; 95% CI 0.62–3.43). Even in patients with tumors >2cm in diameter, the risks of recurrence (HR = 0.82; 95% CI 0.31-2.16) or death (HR = 1.01; 95% CI 0.35-2.95) were not higher for laparoscopic radical hysterectomy than for open radical hysterectomy. The laparoscopic radical hysterectomy and open radical hysterectomy group had 5-year recurrence-free survival rates of 92.8% and 94.4%, respectively (P=0.499). In the meta-analysis of Wang et al. (12), patients who underwent laparoscopic hysterectomy had a lower incidence of postoperative adverse reactions. And the 5-year overall survival (HR 0.91, 95% CI 0.48-1.71; p=0.76) and 5-year disease-free survival (hazard ratio [HR] 0.97, 95% CI 0.56–1.68; p = 0.91) rates of the two groups were not significant different. However, these studies were all retrospective studies and the simple size of the studies included in the meta-analysis were too small. Another meta-analysis regarding laparoscopic hysterectomy and open surgery in 2015 by Cao et al. (13) reached the same conclusion. However, there was only one randomized controlled trial with 30 patients included. The other 21 studies were all retrospective or prospective studies with a small simple size and a short follow-up time. In general, the above researches showed that compared with open surgery, laparoscopic hysterectomy did not reduce the overall survival and disease-free survival (14, 15), but also had the advantages of a decrease in operative blood loss, a shorter hospital stay, and a lower rate of postoperative complications than open radical hysterectomy (12, 13, 16). However, these retrospective studies may have a bias of case mismatch. Because in clinical practice, surgeons will choose cases with earlier stage or smaller lesions to perform laparoscopic hysterectomy. For the other more difficult cases, open surgery was their preferred choice. In November 2018, the New England Journal of Medicine published two results on the comparison of open surgery and minimally invasive surgery. One was a multi-center, prospective, randomized controlled clinical trial (LACC) (1), and the other was a retrospective epidemiological study (2). These two studies compared the recurrence and survival results of patients with early cervical cancer undergoing open surgery and minimally invasive surgery, and found that the survival of patients in the open surgery group was significantly better than that of the minimally invasive surgery group, which completely contrary to previous results (10–16). The same results were obtained in subsequent studies by other institutions (17, 18). It caused great shock and controversy in the international community. Therefore, it can be speculated that the risk factors for early cervical cancer may have changed under the new surgical method of minimally invasive surgery. Although the meta-analysis by Cao et al. (13) has balanced various risk factors, the results of this analysis did not reflect the adverse effects of laparoscopic hysterectomy caused on survival of cervical cancer. Based on previous researches’ conclusions, the risk factors for early stage cervical cancer after minimally invasive surgery have not been fully explored in the past thirty years. Whether the Sedlis criteria is still suitable for early stage cervical cancer is worthy of further thinking and exploration.

In our study, patients who underwent laparoscopic hysterectomy of cervical cancer had a lower 3-year LRFS than the other two groups. The result is the same as the Laparoscopic Approach to Cervical Cancer (LACC) Trial (1). The 62 patients in group B did not fully comply with the Sedlis criteria. As long as there were single or two intermediate risk factors, they all received the treatment of laparoscopy + postoperative radiotherapy, and obtained the same survival as open surgery group, indicating that for patients with early cervical cancer, it is not enough to use Sedlis criteria to assess risk factors and guide postoperative adjuvant treatment. Adjuvant treatment is necessary for early cervical cancer after laparoscopic hysterectomy for patients with early stage cervical cancer. At the same time, our result also confirms from the side that the conclusion of LACC Trial that minimally invasive radical hysterectomy was associated with lower rates of disease-free survival and overall survival than open abdominal radical hysterectomy may be still applicable to “low-risk” patients with early stage cervical cancer. The indications for postoperative adjuvant treatment are still being explored, but a complete consensus has not yet been formed. Different regions still use different standards. Japan Society of Gynecologic Oncology (JSGO) (19) guidelines recommends that patients with one or more intermediate risk factors can be treated with radiotherapy. However, the establishment of JSCO standards did not recognize the negative impact of laparoscopy on survival, and its use has the risk of overtreatment. The European Society of Medical Oncology (ESMO) (20) recommends that patients with intermediate risk factors for early stage cervical cancer do not need any adjuvant treatment after surgery (evidence level 2B). However, some scholars believe that a single intermediate risk factor will not increase the risk of cervical cancer recurrence, but when these intermediate risk factors are combined, the risk of recurrence can increase by 15% to 20% (21). The result of the Laparoscopic Approach to Cervical Cancer Trial has brought further challenges to the establishment of current postoperative adjuvant treatment standards. In terms of 3-year DMFS rates, there were not significant between-group difference among three groups. The results were similar to some previous studies (11, 22, 23). Postoperative radiotherapy did not reduce the rate of distant metastasis, which may be related to the biological characteristics of the tumor. Straume O and Dumoff KL et al. (24, 25) found that lymphatic vessel density was an important indicator of the prognosis of stage I cervical cancer and a low podo-planin immune-reactivity was associated with lymphatic invasion and lymph node metastasis of cervical cancer. Krishnan J et al. (26) found that VEGF-C and VEGF-D were involved in mediating the direction of tumor cell migration. In subgroup analysis, patients with stage IB cervical cancer had a higher rate of local control and overall survival after postoperative radiotherapy, revealing that patients with IB stage cervical cancer may be the part of population who benefited from postoperative radiotherapy after laparoscopic hysterectomy. However, no benefits existed in patients with stage IA and IIA1 disease. The possible reasons may be: The sample size was too small to assess the overall survival benefit. The follow-up time of some patients was not long enough to show the differences on OS or DMFS. Furthermore, the 3-year overall survival was already too high to show a between-group difference in subgroup of stage IA disease. It is necessary to further expand the enrolled population and carry out long-term follow-up.

In addition, the main adverse reactions were grade 1-2 acute hematological toxicity and gastrointestinal reactions for patients receiving adjuvant radiotherapy. The study by Porte lance L (27) showed that the dose distribution for cervical cancer using IMRT was significantly better than those using conventional radiotherapy. In Lin Y et al.’s (28) meta-analysis, the incidence of genital tract adverse reactions and grade 3-4 adverse reactions in in women receiving intensity-modulated radiotherapy were significantly lower than those receiving conventional radiotherapy or three-dimensional conformal radiotherapy. Therefore, for patients with early stage cervical cancer, postoperative radiotherapy with intensity-modulated technology is safe and effective.

Laparoscopic hysterectomy has been widely used in the past thirty years. Due to its negative impact on survival in the LACC Trial, it is no longer recommended in some cervical cancer guidelines. However, we can’t completely deny its clinical benefits. And now laparoscopic hysterectomy is still widely used in other tumors, and whether its negative impact exists in the treatment of other tumors remains to be further investigated. Therefore, we should further explore the mechanism of its negative impact on survival, and develop a more suitable adjuvant treatment standards to bring patients better survival and quality of life at the same time.

In conclusion, there is a risk of local failure in laparoscopic hysterectomy for early stage cervical cancer. Adjuvant radiotherapy can reduce the risk of recurrence and improve local control for women with early cervical cancer and bring survival benefits for patients with stage IB disease after minimally invasive hysterectomy.

## Data Availability Statement

The original contributions presented in the study are included in the article/supplementary material. Further inquiries can be directed to the corresponding author.

## Author Contributions

W-qL and Y-pW designed this study. X-wJ, X-mX, H-yY, X-dL, H-jZ, XO, and Q-lZ collected data and followed the patients. D-jL, Q-fL, and Y-tX performed the data analyses. JL, Q-hD, and Y-xG wrote the paper. W-qL and JL inspected the manuscript critically and took part in the revision of manuscript. All authors contributed to the article and approved the submitted version.

## Funding

The Scientific Foundation of the Second Affiliated Hospital of Guangxi Medical University (Grant/Award Number: EFYKY2020018&2020008).

## Conflict of Interest

The authors declare that the research was conducted in the absence of any commercial or financial relationships that could be construed as a potential conflict of interest.

## Publisher’s Note

All claims expressed in this article are solely those of the authors and do not necessarily represent those of their affiliated organizations, or those of the publisher, the editors and the reviewers. Any product that may be evaluated in this article, or claim that may be made by its manufacturer, is not guaranteed or endorsed by the publisher.
